# Novel Multilocus Sequence Typing and Global Sequence Clustering Schemes for Characterizing the Population Diversity of Streptococcus mitis

**DOI:** 10.1128/jcm.00802-22

**Published:** 2022-12-14

**Authors:** Akuzike Kalizang’oma, Brenda Kwambana-Adams, Jia Mun Chan, Aishwarya Viswanath, Andrea Gori, Damien Richard, Keith A. Jolley, John Lees, David Goldblatt, Sandra Beleza, Stephen D. Bentley, Robert S. Heyderman, Chrispin Chaguza

**Affiliations:** a NIHR Mucosal Pathogens Research Unit, Division of Infection and Immunity, University College London, London, United Kingdom; b Malawi-Liverpool-Wellcome Programme, Blantyre, Malawi; c UCL Division of Medicine, University College London, London, United Kingdom; d UCL Genetics Institute, University College London, London, United Kingdom; e Department of Zoology, University of Oxford, Oxford, United Kingdom; f Pathogen Informatics and Modelling, European Bioinformatics Institute, Hinxton, United Kingdom; g University College London, Great Ormond Street Institute of Child Health, London, United Kingdom; h University of Leicester, Department of Genetics and Genome Biology, Leicester, United Kingdom; i Parasites and Microbes, Wellcome Sanger Institute, Hinxton, United Kingdom; j Department of Epidemiology of Microbial Diseases, Yale School of Public Health, Yale University, New Haven, Connecticut, USA; k Department of Clinical Infection, Microbiology and Immunology, University of Liverpool, Liverpool, United Kingdom; l Yale Institute for Global Health, Yale University, New Haven, Connecticut, USA; National Institute of Allergy and Infectious Diseases

**Keywords:** *Streptococcus mitis*, multilocus sequence typing, PopPUNK sequence clustering, population diversity, within-host diversity, transmission

## Abstract

Streptococcus mitis is a common oral commensal and an opportunistic pathogen that causes bacteremia and infective endocarditis; however, the species has received little attention compared to other pathogenic streptococcal species. Effective and easy-to-use molecular typing tools are essential for understanding bacterial population diversity and biology, but schemes specific for S. mitis are not currently available. We therefore developed a multilocus sequence typing (MLST) scheme and defined sequence clusters or lineages of S. mitis using a comprehensive global data set of 322 genomes (148 publicly available and 174 newly sequenced). We used internal 450-bp sequence fragments of seven housekeeping genes (*accA*, *gki*, *hom*, *oppC*, *patB*, *rlmN*, and *tsf*) to define the MLST scheme and derived the global S. mitis sequence clusters using the PopPUNK clustering algorithm. We identified an initial set of 259 sequence types (STs) and 258 global sequence clusters. The schemes showed high concordance (100%), capturing extensive S. mitis diversity with strains assigned to multiple unique STs and global sequence clusters. The tools also identified extensive within- and between-host S. mitis genetic diversity among isolates sampled from a cohort of healthy individuals, together with potential transmission events, supported by both phylogeny and pairwise single nucleotide polymorphism (SNP) distances. Our novel molecular typing and strain clustering schemes for S. mitis allow for the integration of new strain data, are electronically portable at the PubMLST database (https://pubmlst.org/smitis), and offer a standardized approach to understanding the population structure of S. mitis. These robust tools will enable new insights into the epidemiology of S. mitis colonization, disease and transmission.

## INTRODUCTION

Streptococcus mitis is an abundant oral commensal that can escape the oral niche to cause invasive disease (1, 2). S. mitis is a dominant cause of infective endocarditis (1), a serious multisystem disease associated with high mortality that approaches 25 to 30% at 1 year postdiagnosis despite optimal treatment (3, 4). Also causing bacteremia among immunosuppressed patients undergoing chemotherapy (5–7), S. mitis infections are often associated with antimicrobial resistance (AMR) (2, 8).

However, understanding S. mitis naso-oropharyngeal carriage, transmission, and disease remains challenging due to the unreliable species identification methods frequently used, which fail to either differentiate S. mitis from other viridans group streptococci or provide a clear picture of population structure (9). For example, early studies investigated S. mitis biovar 1 genetic diversity using ribotyping, which uses restriction endonuclease to digest DNA followed by separation of the digested DNA fragments by electrophoresis on agarose gel (10–12). Other nonculture methods were also used to define genotypes based on sequencing of a limited number of genes such as the single glucose-6-phosphate dehydrogenase gene (13). Both nonculture methods suggested extensive diversity of S. mitis between and within individuals (10, 12, 13); however, accurate identification to the species level is uncertain as Streptococcus oralis isolates have often been misidentified as S. mitis biovar 1 (14).

Several pioneering S. mitis studies that relied on traditional microbiology and biochemistry methods (14–17) may have misidentified S. mitis (18), and previous studies have highlighted the challenges in accurately differentiating S. mitis and S. oralis based on the analysis of 16S rRNA gene amplicon sequencing data (19). More recently, matrix-assisted laser desorption ionization–time of flight mass spectrometry (MALDI-TOF MS) has also shown low precision in differentiating S. mitis from other closely related species (20, 21). A combination of the culture-based methods and whole-genome sequencing (WGS) offers an unambiguous method for identifying bacterial species and defining population structure with high resolution (22) and has distinguished clinical strains of S. mitis from other mitis group streptococci with high accuracy (23).

Although the application of WGS for analysis of population structure has provided insights into the genomic epidemiology for numerous bacterial species (24–27), standard bioinformatics tools and molecular schemes for characterizing S. mitis genetic diversity lag behind other clinically relevant bacterial species. These tools and schemes include multilocus sequence typing (MLST), which provides a simple and portable method for identifying bacterial clones, typically using seven housekeeping genes (28, 29). To date, MLST schemes have been defined for 130 bacterial species (29), including 12 Streptococcus species, such as Streptococcus pneumoniae (29, 30).

To complement MLST and other molecular typing approaches, newer methods, such as PopPUNK, have been developed, which takes into account variation in both core and accessory genomes to population structure (31). The PopPUNK algorithm is applicable to different species, able to distinguish very similar strains at a high resolution, and generates reproducible sequence clusters (31). This is in contrast to previously widely used methods, such as Bayesian Analysis of Population Structure (BAPS) (32, 33), which did not maintain consistent cluster naming upon integration of new strain data, which resulted in ambiguous definition of bacterial clades between laboratories (31). The application of PopPUNK to study S. pneumoniae has revealed global pneumococcal lineages and facilitated surveillance of carriage and disease strains, AMR, and the impact of serotype-specific conjugate vaccines (34).

The availability of a dedicated MLST scheme and PopPUNK sequence clusters for S. mitis would provide a standard method to define its population structure and to understand its genetic diversity and epidemiology.

Here, we describe the development and application of MLST and PopPUNK sequence clustering schemes for S. mitis to a comprehensive data set of confirmed publicly available and newly sequenced S. mitis isolates obtained from multiple sources and clinical contexts. Overall, our findings showed high concordance of the methods and captured the extensive genetic diversity of the S. mitis species at both individual and population levels, highlighting the utility of these methods for understanding the population genetics and epidemiology of this neglected bacterial species.

## MATERIALS AND METHODS

### Genome selection and identification to the species level.

A comprehensive data set of publicly available S. mitis genomes was obtained together with additional isolates sequenced from a carriage study (see below) to develop the S. mitis MLST and global genomic clustering schemes (see Table S1 in the supplemental material). Genome quality was determined using the quality assessment tool for genome assemblies (QUAST v.5.0.2) using default settings (35). Taxonomic classification of S. mitis was initially done using KRAKEN v.1.0 (22) against the MiniKraken DB_8GB database. Default settings were used, and the taxonomic labels were converted to reports using KRAKEN postprocessing scripts (https://ccb.jhu.edu/software/kraken/). Species-level identification was also confirmed using KRAKEN v.2.1.2 (36) against the minikraken2_v2_8GB_201904 database. Default settings were used, and species-level reports were generated on running the initial command. The online PathogenWatch Speciator tool for assigning species to an assembled genome was also used (https://pathogen.watch/), and the in-house species identification tool applied MASH to search a curated NCBI RefSeq database. Finally, GTDB-Tk v.2.1.0 (37) was used together with the reference data version r207 to classify the species. Default parameters were used following the GTDB-Tk manual. Genomic analyses were conducted using Wellcome Sanger Institute (WSI) and University College London (UCL) computing clusters.

### Development of the S. mitis MLST scheme.

To define the MLST scheme, S. mitis genomes were first annotated using Prokka v.1.13.4 (38), and pangenome analysis was then conducted using the annotated S. mitis genomes to obtain core genes present in 100% of the isolates using Panaroo v.1.2.8 (39). The core genes were screened to identify and select 7 housekeeping genes evolving under negative selection. GenomegaMap (40) was used to determine the evolutionary pressures on protein-coding regions (nonsynonymous/synonymous substitution [*dN*/*dS*] ratios) for core genes with names and known functions. Seven housekeeping genes evolving under negative selection (*dN*/*dS* ratio of <1) were selected for the MLST scheme, and 450-bp internal fragments for each gene were used to define gene alleles. The seven genes were also selected for consistency with most of the MLST schemes. The MLST genes were analyzed within the Molecular Evolutionary Genetics Analysis (MEGA) software v.10.0 (41) and visualized on the S. mitis B6 reference genome (GenBank accession no. GCA_000027165.1) using DNAPlotter v.18.2.0 (42). Nucleotide-BLAST v.2.10.1 was also used to rule out duplication of the seven housekeeping genes among confirmed S. mitis genomes. Unique alleles were assigned arbitrary numbers, and the sequence type (ST) was determined by the combination of alleles at the seven housekeeping gene loci (*accA*, *gki*, *hom*, *oppC*, *patB*, *rlmN*, and *tsf*). Simpson’s diversity index (SDI) was used to measure the diversity of the alleles for each MLST gene using an online tool (http://www.comparingpartitions.info/) (43). The MLST software (https://github.com/tseemann/mlst) was then used together with the S. mitis scheme to define STs of S. mitis isolates (29), and the BIGSdb BURST analysis tool was used to group related STs (29).

### Inference of population structure.

An alignment of polymorphic sites was generated from the core genome alignment using Snp-Sites v.2.5.1 (44). The isolates were clustered into subpopulations or sequence clusters (SCs) using hierBAPS as part of the Bayesian analysis of population structure (BAPS) v.6.0 software (32, 33).

### Establishing global S. mitis sequence clusters.

S. mitis global sequence clusters were established by applying PopPUNK on the S. mitis genome data set. Core and accessory genomes were determined using default PopPUNK parameters (31); however, the maximum thresholds for core and accessory distances were increased due to extensive S. mitis diversity that was incorrectly identified as contamination for all genomes in the data set. The Bayesian-Gaussian mixture model was applied and refined using the core and accessory distances as it best suited the population structure of the data set.

### Application of genomic tools to S. mitis carriage isolates.

A prospective pilot carriage study was conducted among healthy adults in November 2019 to obtain S. mitis isolates that would test the robustness and discriminatory power of the developed genomic tools. Healthy adults above the age of 18 who were UCL students or staff were eligible. Current or recent antibiotic use within 2 weeks prior to sampling was an exclusion criterion. In total, 12 healthy adults were recruited.

Briefly, nasopharyngeal, oral, and oropharyngeal samples were obtained. Nasopharyngeal swab samples were taken with Copan FLOQswabs (Copan, USA) using a previously described technique (45). Oral swab samples were taken using Copan FLOQswabs, and to ensure adequate sampling of regions known to be colonized by S. mitis (12), the surface of the teeth, dorsum of the tongue, hard palate, left and right buccal mucosa, and maxillary and mandibular gingiva were sampled with the same swab. Both oral and nasopharyngeal swabs were stored in skim milk-tryptone-glucose-glycerol (STGG) medium. Cough samples were obtained from volunteers directly onto sterile Columbia blood agar (CBA) plates. Additional respiratory samples were collected using a facemask device with a soluble polyvinyl alcohol (PVA) inner strip as previously described (46), and PVA strips were dissolved in Todd-Hewitt broth with yeast extract (THY) culture medium prior to inoculation.

### Microbiology and molecular screening.

Volumes of 10 μL of STGG and THY were used to inoculate CBA plates, which were incubated together with the direct cough plates for 18 h in 5% CO_2_ and at 37°C. Alpha-hemolytic colonies were tested for optochin resistance, and a maximum of 6 alpha-hemolytic colonies were subcultured. DNA was obtained from purified isolates using heat lysis, screened using the S. mitis *pheA* PCR primers, and visualized via electrophoresis as previously described (47) to obtain potential S. mitis isolates. S. mitis NCTC 12261 (PHE, United Kingdom) was used as the positive control.

### DNA extraction and whole-genome sequencing.

Extracted genomic DNA for whole-genome sequencing (WGS) was obtained from pure overnight plate cultures that were *pheA* PCR positive using the Qiagen DNeasy blood and tissue kit and following the manufacturer’s instructions (Qiagen, Germany). DNA quantification using Qubit fluorometric quantification (Thermo Fisher, United Kingdom), genomic DNA library preparation, and WGS were carried out by the UCL Pathogen Genomics Unit (PGU). The Illumina NextSeq platform (Illumina, San Diego, CA, USA) was used for WGS, which generated paired-end sequence reads of 150 bp in length and 50 to 100× coverage.

### Genome assembly.

Illumina sequencing reads were checked for quality using FastQC (https://www.bioinformatics.babraham.ac.uk/projects/fastqc/). Sequenced read adapter trimming was done using Trimmomatic v.0.39 (48), and a phred score of at least 33 per read was used as the minimum quality score threshold. *De novo* genome assembly was performed using SPAdes v.3.12 (49), and genome quality was determined using the quality assessment tool for genome assemblies (QUAST) v.5.0.2 (35).

### Sequence mapping and pairwise SNP distances.

Genetic diversity of S. mitis isolated within and between the volunteers was investigated through mapping of sequence reads, single nucleotide polymorphism (SNP) distances calculated between isolates, and phylogenetic reconstruction. Snippy v.4.6.0 (https://github.com/tseemann/snippy) was used to map confirmed S. mitis sequence reads to the S. mitis B6 reference genome (GenBank accession no. GCA_000027165.1) and obtain a core genome SNP alignment, which was used to determine genetic diversity. Pairwise SNP distances were also calculated between the S. mitis strains using snp-dist v.0.7.0 (https://github.com/tseemann/snp-dists) and the S. mitis SNP alignment. The pairwise SNP distance matrix was visualized as a heat map in R v.2.11.1 (50).

### Phylogeny and population diversity.

The S. mitis SNP alignment of polymorphic sites was then used to construct maximum likelihood phylogenies using fasttree v.2.1.10 (51). The generalized time-reversible model of nucleotide evolution was used to generate the phylogenies, which were then visualized and annotated using the online Interactive Tree of Life (iToL) software v.3.0 (52). Isolates were then clustered into subpopulations using hierBAPS as part of the Bayesian analysis of population structure (BAPS) v.6.0 software (32), STs (29), and PopPUNK global sequence clusters (31), which were then visualized in microreact v.5.93.0 (53).

### Antimicrobial resistance genotyping.

AMR genes were identified among the S. mitis isolates using ARIBA v.2.14.6 (54), and the ResFinder database was used as a reference for AMR genes (55).

### Experimental validation of the S. mitis MLST scheme.

Amplification primers for the seven MLST genes (*accA*, *gki*, *hom*, *oppC*, *patB*, *rlmN*, and *tsf*) were designed using Primer3 (56). *In silico* PCR using the ipcress tool v.0.8.3 was first used to predict amplification for all S. mitis genomes, closely related species, and non-Streptococcus species. Default settings were used for *in silico* PCR amplifications (https://www.ebi.ac.uk/about/vertebrate-genomics/software/ipcress-manual). Thirty (*n* = 30) S. mitis isolates were then selected for experimental validation of the MLST scheme, where isolates were selected based on the rarity of alleles and the isolation condition. S. mitis NCTC 12261 (SK142; type strain) was used as a positive control (57). Selected isolates are listed in Table S1.

The 7 MLST internal fragments were amplified from genomic DNA using a commercially available PCR master mix per the manufacturer’s instructions (OneTaq Quick Load 2× master mix; NEB). The primers are listed in Table S2, and the PCR cycling conditions used are as follows: 1 cycle of denaturation at 94°C for 30 s, followed by 35 cycles of amplification at 94°C for 15 s, 54°C for 15 s, and 68°C for 30 s, and then 1 cycle of final extension at 68°C for 5 min. Further optimization of the annealing temperature may be required to ensure that only a single DNA fragment is sequenced. PCR products were purified and Sanger sequenced in both directions by LGC Genomics. The paired sequences were merged using EMBOSS merger (58) and visualized in MEGAX, and PubMLST (https://pubmlst.org/smitis) was used to define alleles and STs (29).

### Ethics.

Ethical approval from UCL Research Ethics Committee (UCL REC) was obtained for the prospective pilot carriage study (UCL REC no. 15101/001). Informed written consent was obtained from all volunteers at the time of recruitment.

### Data availability.

Genome sequences generated in this project are available under BioProject no. PRJEB55310. Publicly available genomes used in this project are available under BioProject no. PRJNA480039, PRJEB42564, PRJEB42963, and PRJEB53188. Accession numbers for all genomes used are also listed in Table S1. MLST nucleotide sequences generated from Sanger sequencing are available under accession no. OP792763 to OP792980.

## RESULTS

### Genome selection and identification to the species level.

Initial taxonomic screening of the genome assemblies using KRAKEN v.1.0 determined that all 322 genomes (100%) were S. mitis. Additional screening using KRAKEN v.2.1.2 also identified S. mitis among 322 genomes (100%) (see Tables S3 to S6 in the supplemental material). Identification to the species level using the MASH approach in PathogenWatch determined 299 out of 322 genomes (93%) were S. mitis (Table S7), while 21 out of 322 (7%) were unclassified Streptococcus species. GTDB-Tk determined 212 out of 322 (66%) were S. mitis (Table S8), while 48 out of 322 (15%) were unclassified Streptococcus species. The taxonomic screening tools use different approaches and reference databases (22, 36, 37); therefore, variation in the results was expected (Table S9). We proceeded with genomes identified using KRAKEN v.1.0 and v.2.1.2 due to consistency and to maximize capturing S. mitis genetic diversity.

### Molecular typing of S. mitis using a novel MLST scheme.

**FIG 1:**
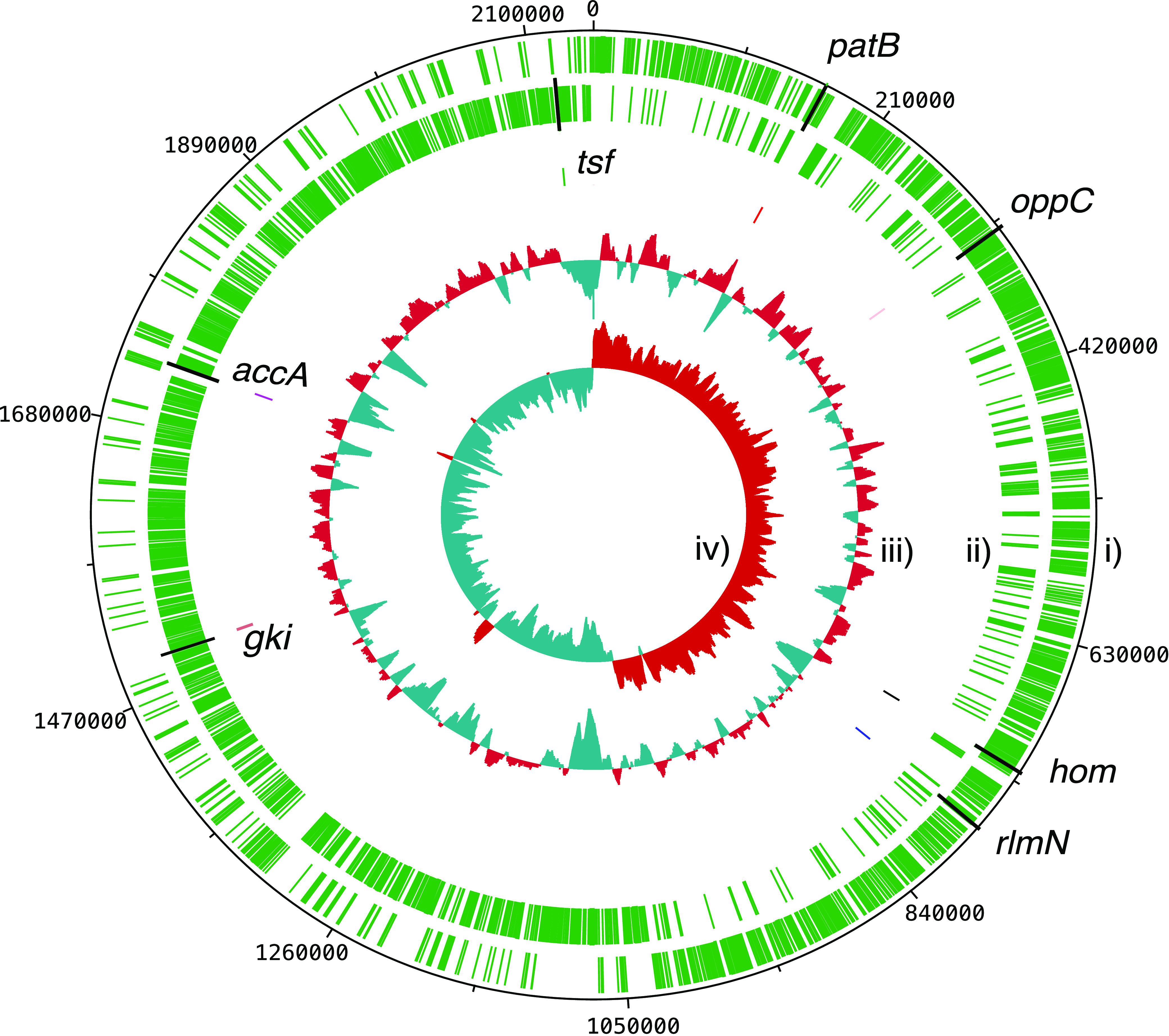
Location of the selected seven MLST genes on the S. mitis B6 reference genome (GenBank accession no. GCA_000027165.1). The plot shows S. mitis B6 as a circular genome, and the genome size in base pairs is shown on the outermost numbered region. The tracks from the outside represent the (i) forward and (ii) reverse coding sequence (CDS), (iii) GC content, and (iv) GC skew. Above average and below average values for GC content and GC skew are shown in red and blue, respectively. The locations of the MLST genes are indicated on the forward or reverse CDS.

We used 322 S. mitis genomes to develop an initial MLST scheme for S. mitis, where assembly quality was established using QUAST (Tables S10 and S11). Among the total 322 genomes, 158 (49.1%) were from carriage, 138 (42.9%) were from infective endocarditis, 13 (4.0%) were from bacteremia, 12 (3.7%) were from unknown sources, and 1 (0.3%) was from pneumonia (Table S1). The genomes included clinically relevant isolates to obtain a scheme that can be applied in the clinical setting. From the total number of 1,183 core S. mitis genes obtained through the pangenome analysis, seven housekeeping genes with established names, known functions, and evolving under negative selection were selected to define the S. mitis MLST scheme: acetyl coenzyme A (acetyl-CoA) carboxylase carboxyl transferase subunit alpha (*accA*), glucose kinase (*gki*), homoserine dehydrogenase (*hom*), ABC transporter permease (*oppC*), pyridoxal phosphate-dependent aminotransferase (*patB*), rRNA large subunit methyltransferase N (*rlmN*), and translation elongation factor Ts (*tsf*). These genes have important roles in lipid metabolism (*accA*), amino acid biosynthesis (*hom*), transmembrane transport (*oppC* and *patB*), protein biosynthesis (*tsf*), glucose metabolism (*gki*), and rRNA and tRNA processing (*rlmN*) (59). The locations of the genes on the S. mitis B6 reference genome (GenBank accession no. GCA_000027165.1) are shown in Fig. 1.

The MLST genes were characterized to determine gene length, GC content, average evolutionary pressures on protein-coding regions (*dN*/*dS*), number of alleles identified, and allele diversity (Table 1). The average *dN*/*dS* ratios for the genes were less than 1, indicating purifying selection making them ideal for use in the typing scheme. Allele diversity calculated using Simpson’s diversity index (SDI) revealed extensive diversity among the alleles as all values were ~1, which suggested that genetic variation in these genes would be sufficient to capture the genetic relatedness of S. mitis isolates.

**TABLE 1:** Genetic characterization of the seven housekeeping genes selected for the S. mitis MLST scheme

Gene	Gene length (bp)	GC content (%)	*dN*/*dS* ratio (ω)	No. of alleles	Simpson’s diversity	95% confidence interval
*accA*	768	43	0.0766	176	0.987	0.982–0.992
*gki*	924	45	0.0511	174	0.983	0.977–0.989
*hom*	1,287	42	0.0655	176	0.984	0.978–0.990
*oppC*	927	35	0.0364	142	0.966	0.956–0.975
*patB*	1,206	41	0.1116	195	0.991	0.987–0.995
*rlmN*	1,104	40	0.0246	184	0.987	0.981–0.992
*tsf*	1,041	41	0.0711	144	0.981	0.974–0.987

**FIG 2:**
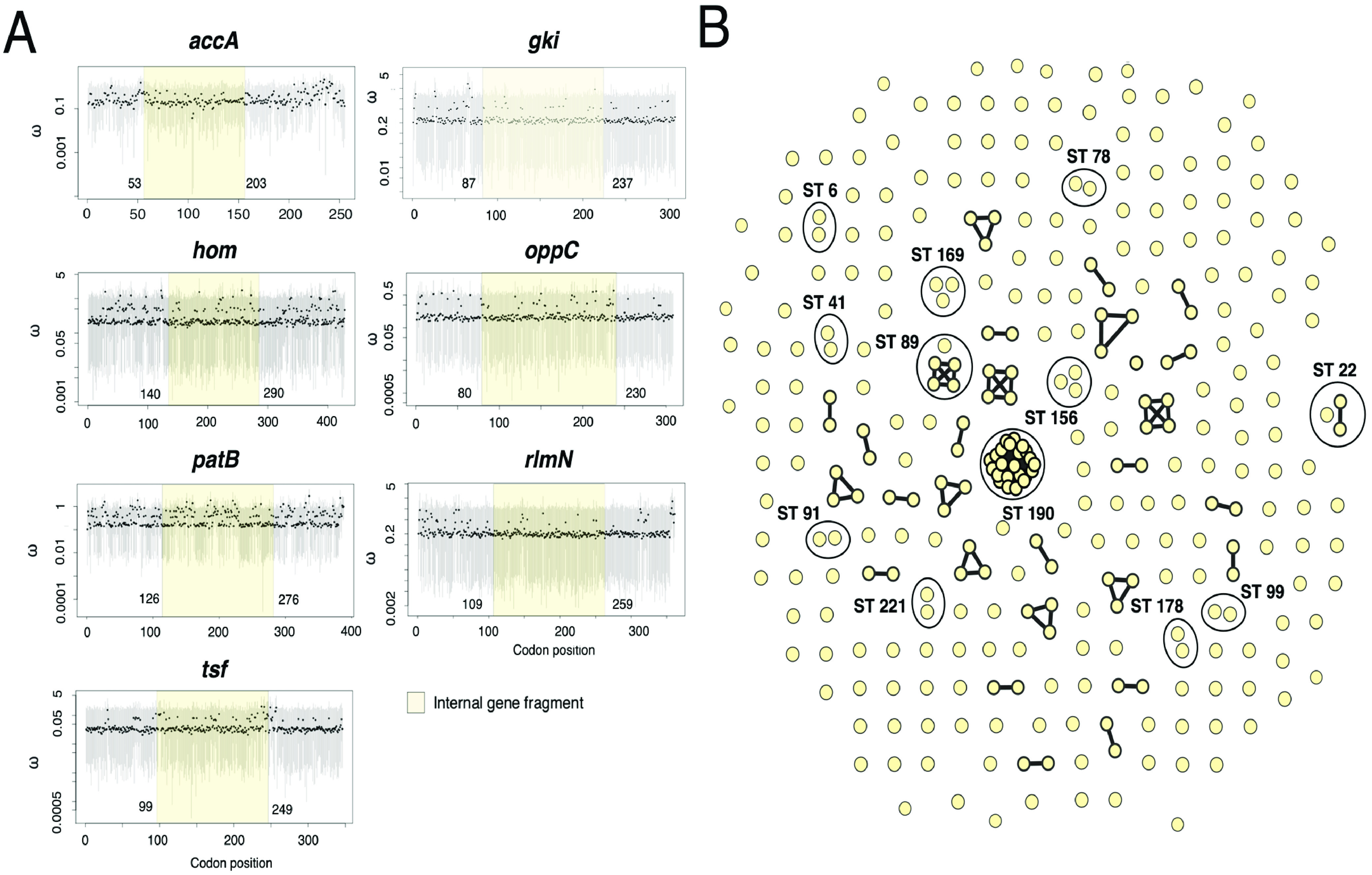
Development and application of the novel S. mitis MLST scheme. (A) Estimates of the *dN*/*dS* (ω) ratios among seven S. mitis housekeeping genes. *dN*/*dS* point estimates determined by GenomegaMap are shown by black points, and 95% credibility intervals are shown by gray bars for codon positions along each gene. The internal fragments selected to define alleles are highlighted. (B) Sequence types and clonal groups identified using the novel S. mitis MLST scheme. Unlinked nodes represent unique STs, while linked nodes belong to the same ST. Isolates assigned to the same clonal group are shown by ellipses, which have been assigned the name of one ST found within the group.

Evolutionary pressures on protein-coding regions (*dN*/*dS* ratios) for the seven housekeeping genes (*accA*, *gki*, *hom*, *oppC*, *patB*, *rlmN*, and *tsf*) were calculated along the entire gene length, including the 450-bp internal fragments used for the scheme, which largely indicated a lack of positive or neutral selection (Fig. 2A). The MLST scheme was then applied to the data set of 322 S. mitis isolates to identify similar and diverse STs, and a total of 259 different STs were resolved. The BIGSdb BURST analysis tool identified 11 groups of closely related STs; however, the founding (ancestral) genotype of the clonal complex could not be predicted due to the low frequency of STs per group. Figure 2B shows the total number of unique STs and clonal groups. MLST allows for the integration of new genomic data; therefore, as additional S. mitis isolates are analyzed more STs and clonal complexes will be identified. The S. mitis MLST scheme is available on PubMLST (https://pubmlst.org/smitis) (29).

### Defining S. mitis global sequence clusters.

To establish the global S. mitis sequence clusters, a pangenome analysis was conducted using the S. mitis data set, and totals of 1,183 core and 9,315 accessory genes were identified. Bayesian Analysis of Population Structure (BAPS) was initially applied to the S. mitis core genome SNP alignment to investigate the population structure of the species. However, BAPS generates inconsistent sequence cluster names and the method could only resolve 6 sequence clusters despite the highly diverse S. mitis data set (Fig. S1); therefore, BAPS was not adopted. In contrast, PopPUNK defines clusters with consistent names when used with the same standard database; therefore, we applied PopPUNK to established global S. mitis sequence clusters.

**FIG 3:**
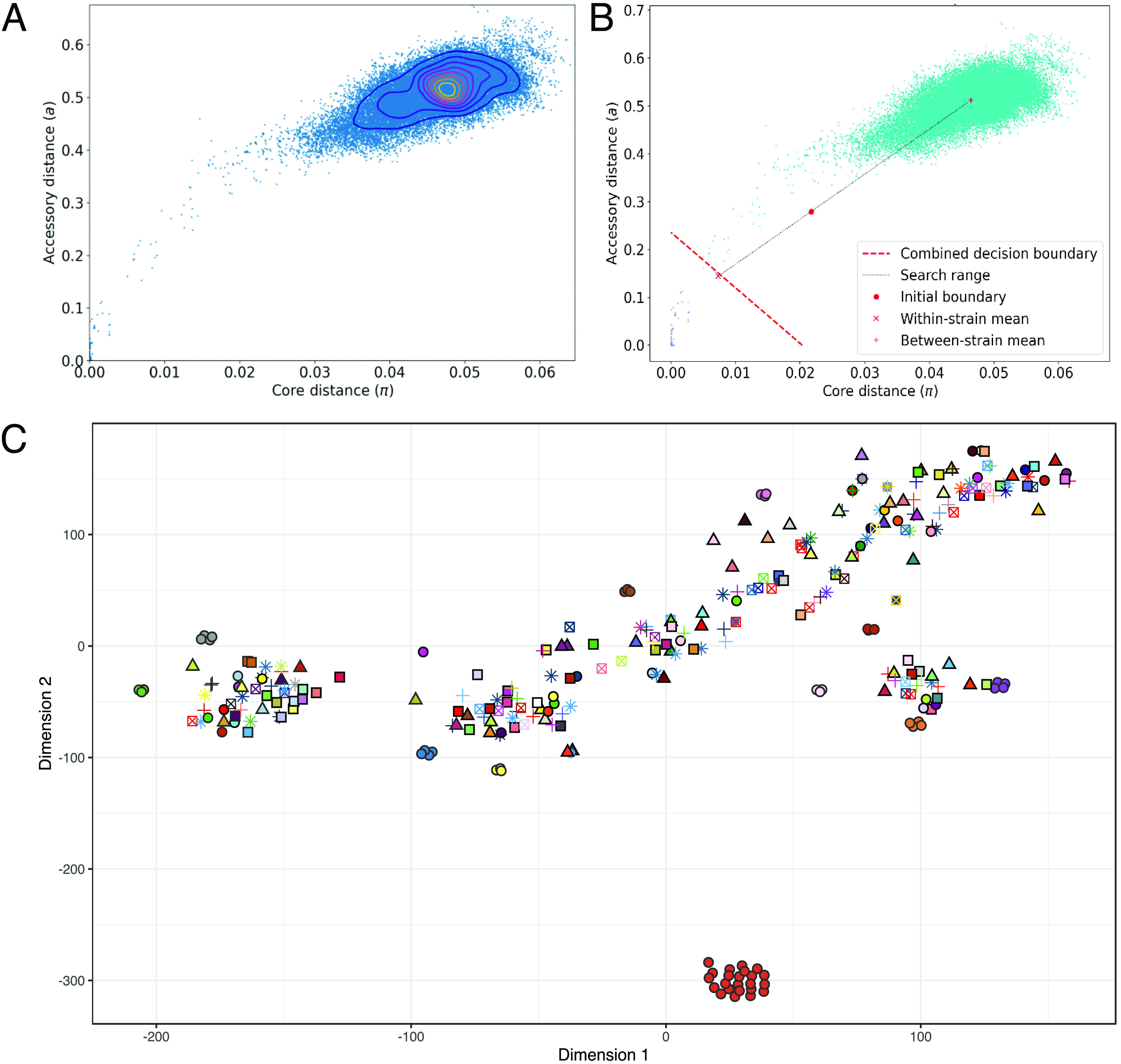
Global sequence cluster analysis of S. mitis genomes using PopPUNK. (A) Scatter plot based on core and accessory distances among all 322 S. mitis genomes in the database using PopPUNK. A node represents an individual isolate. (B) PopPUNK model fitting and refinement using the two-dimensional Gaussian mixture model. The threshold or combined decision boundary for defining within-strain relationships is then refined using a network score in order to generate a sparse but highly clustered network. (C) Accessory distances of S. mitis genomes produced using t-SNE (71). Isolates of the same color and shape that group together demonstrate the sequence clustering algorithm of PopPUNK using the global S. mitis data set.

The population of S. mitis is represented by a network where a node represents an individual isolate using PopPUNK (Fig. 3A), and nodes that are closer to the origin represent within-strain relationships (Fig. 3B). Comparison of core and accessory genetic distances among S. mitis genomes in the data set revealed that isolates were highly diverse as shown by clustering based on greater core and accessory genetic distances (Fig. 3A and B), with a few highly similar isolates that were likely from the same individuals sampled in the small cohort with lesser core and accessory genetic distances. A large cluster of 23 isolates from global cluster 1 were from the United States (NCBI repository); however, isolate metadata were unavailable; therefore, it was unknown whether the isolates were from the same individual or individuals living within close proximity to each other. Overall, PopPUNK identified 258 global sequence clusters, and the genetic distances based on the accessory genomes are shown in Fig. 3C. The PopPUNK S. mitis reference database for inferring S. mitis global sequence clusters is publicly available (https://poppunk.net/).

### High degree of within- and between-host S. mitis genetic diversity among carriage isolates confirmed by MLST.

**FIG 4:**
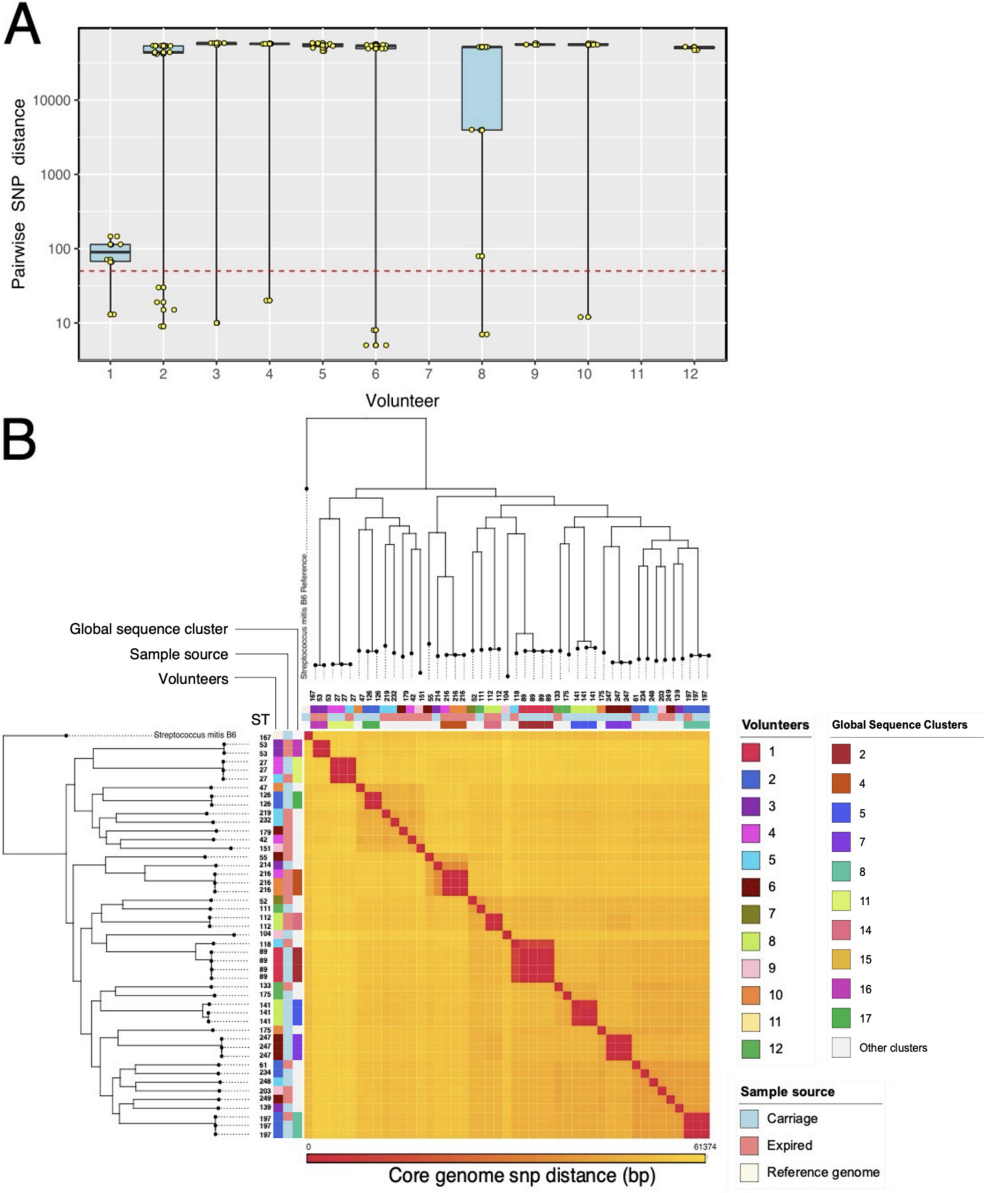
Within- and between-host genetic diversity of S. mitis isolates using pairwise SNP distances and MLST. (A) Pairwise SNP distances of S. mitis isolates obtained from carriage and expired respiratory secretions. The strip charts and box plots show the number of SNPs calculated between isolates from 10 volunteers with 2 or more sequenced S. mitis isolates. The *y* axis is shown in log_10_ scale for clarity. The red dotted horizontal line marks the threshold for what we considered the same strain (<50 bp). (B) Phylogeny and heat map of SNP differences between S. mitis isolates. Pairwise SNP distances were interpreted as a heat map using the R statistical program with heat map clustering methods. Maximum likelihood SNP phylogenies across the top and the left of the heat map indicate the relatedness of the isolates. Isolate ST, volunteer origin, sampling source, and global sequence cluster are adjacent to phylogeny. SNP distances ranged from 0 to 61,374 bp and correspond to a color gradient ranging from red (lowest distance value) to yellow (highest distance value).

To demonstrate the ability of the genomic tools to resolve S. mitis genetic diversity, we applied the tools through the prospective pilot carriage study that sought to assess S. mitis genotypes that are carried among a cohort of 12 healthy volunteers (age range, 18 to 60 years). We obtained a total of 49 confirmed S. mitis isolates from the volunteers and sequenced a median of 4 genomes per individual (range, 1 to 7). Sequencing quality is shown in Fig. S2, and contamination was checked among the assemblies as previously described (Tables S5 and S6). Since the calculation of the pairwise SNP analysis required 2 or more isolates, we excluded volunteers 7 and 11 as they had only one sequenced S. mitis isolate each. The median number of SNPs between pairs of isolates among the 10 volunteers with more than one S. mitis isolate ranged from 92 to 58,466 bp (Fig. 4A). We considered isolates with an SNP distance of <50 bp to be the same strain, and therefore 7 out of 10 volunteers each had one or more isolates that were potentially the same strain. Apart from volunteer 1, who had isolates that were all closely related based on SNP distance (13 to 146 SNPs), the majority of volunteers with more than one isolate (9 out of 10) had diverse isolates with median pairwise SNP distances of more than 50,000 bp, which suggested high within-host genetic diversity.

We next constructed a maximum likelihood phylogenetic tree and pairwise SNP distance matrix from the whole-genome sequence alignment of the isolates to investigate within- and between-host genetic diversity (Fig. 4B). We applied the devised S. mitis MLST scheme to determine STs for the S. mitis isolates. In total, we found 31 unique STs among the 49 S. mitis isolates obtained from carriage and shedding, which suggested a highly genetically diverse S. mitis population among asymptomatic carriers. Each sampled individual carried a median of three unique STs, but the number of STs ranged from 1 to 5. The STs determined by the S. mitis MLST scheme were supported by pairwise SNP distances as few pairwise SNP distances between isolates were observed for the same ST (Fig. 4A). For example, the four S. mitis STs isolated from volunteer 1 were all ST89, with a median pairwise SNP distance of 92 bp. A high degree of within-host genetic diversity was also supported by MLST and pairwise SNP distances, both methods that demonstrate the presence of multiple S. mitis lineages within the same host. For example, MLST identified multiple STs in volunteer 2, namely, ST126, ST61, ST234, and ST197, which had a mean pairwise SNP distance of 43,888 bp (Fig. 4B). The S. mitis MLST scheme also identified the same ST in different individuals. These STs included ST216 isolated from volunteers 4, 10, and 11 and ST27 isolated from volunteers 5 and 4. We found pairwise SNP distances of 21 to 23 bp and 11 to 20 bp for the ST216 and ST27 isolates sampled from different individuals, respectively, which suggested potential recent direct or indirect transmission events.

### High concordance of global sequence clusters, sequence types, and AMR profiles.

**FIG 5:**
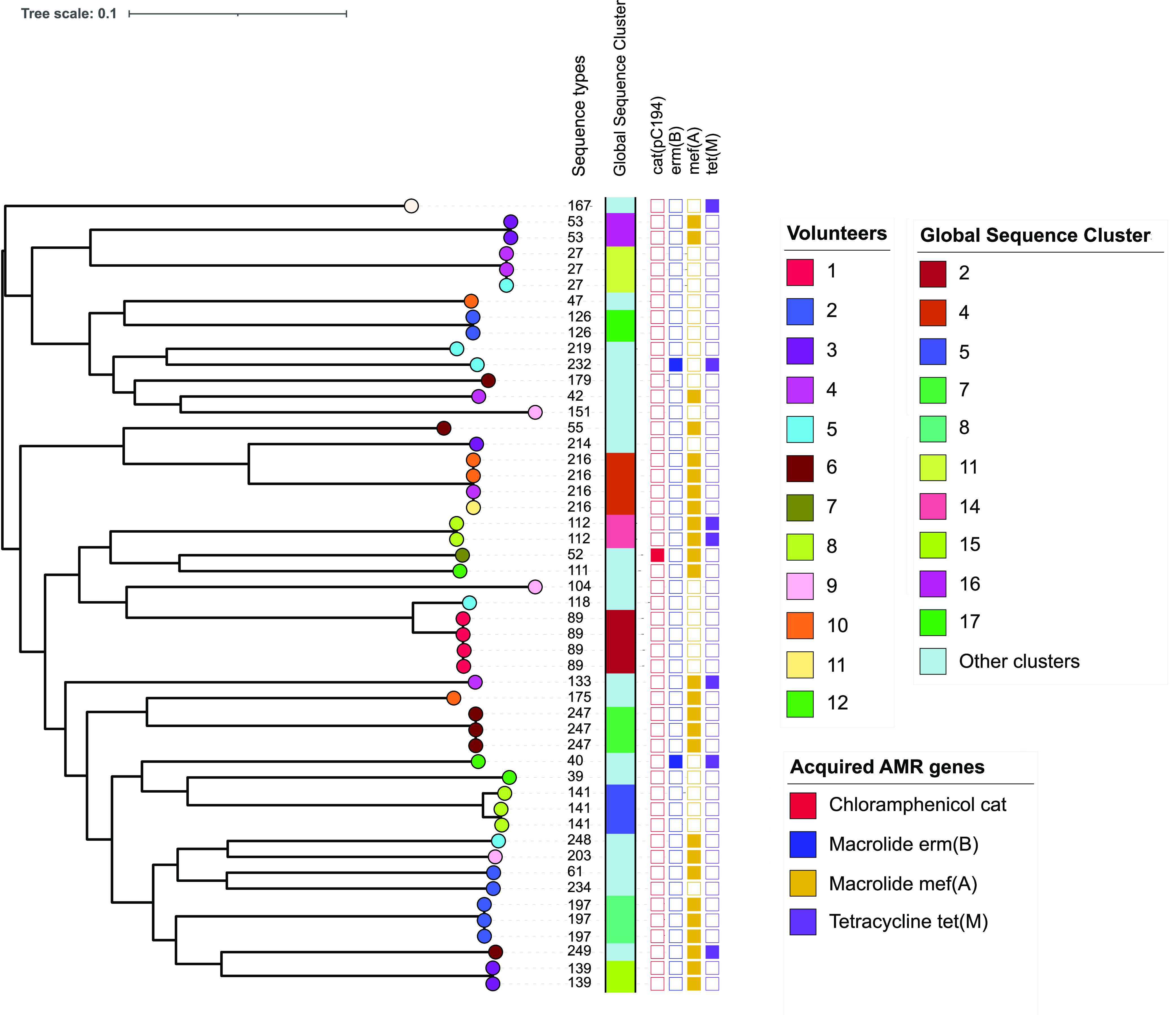
Maximum likelihood core genome phylogeny of S. mitis isolates obtained from healthy volunteers. The ML phylogeny of S. mitis isolates was constructed using SNPs from a mapping alignment of sequencing reads to S. mitis B6, and the tree was visualized in iTOL. The ML phylogeny demonstrates genetic similarity and diversity among the S. mitis isolates. The colored tips of the phylogeny show the volunteers, and other isolate metadata, namely, ST, global sequence cluster, and antimicrobial resistance genes, are shown. The tree was rooted at the midpoint of the branch separating the most genetically diverse genomes.

We investigated the genetic diversity of the sampled S. mitis isolates by constructing a phylogenetic tree and clustering the isolates into genetically distinct subpopulations. The maximum likelihood phylogenetic tree generated from mapped reads showed that the 49 S. mitis isolates obtained from the healthy individuals clustered into 31 global sequence clusters corresponding to 31 STs (Fig. 5), which illustrated a high concordance between the two typing schemes. We then determined antimicrobial resistance profiles to further support the observations of specific STs and sequence clusters. We identified a total of 4 acquired AMR gene classes among the 49 S. mitis isolates through mapping sequenced reads against reference genes from the ResFinder AMR database. Identified AMR genes included those that confer resistance to chloramphenicol, macrolide, and tetracycline antibiotics. The AMR genes were also concordant among isolates of the same ST and sequence cluster. These findings suggested that the MLST and global sequence clustering schemes were robust, and their integration in WGS analysis pipelines would ensure standardized molecular typing of S. mitis isolates to identify similar groups of isolates at local and global scales.

### Validation of the S. mitis MLST scheme.

We validated the S. mitis MLST scheme using *in silico* and conventional PCR methods. *In silico* amplification for the 7 MLST genes was observed for all 322 S. mitis isolates using the designed primers (Table S2). Among the 19 different species screened, *in silico* amplification for all 7 MLST genes was observed for S. mitis, S. pneumoniae, and *S. pseudopneumoniae* reference genomes (Table S12), which was supported by conventional PCR (Fig. S3). While biochemical and molecular testing can contribute to differentiating S. pneumoniae from other closely related species (45, 60), we recommend the use of whole-genome sequencing and bioinformatic species-level determination to accurately identify S. mitis due to the high genetic similarity with other closely related mitis group species.

We carried out Sanger sequencing for the amplified PCR products of 30 S. mitis isolates obtained from carriage and disease, and we compared the alleles obtained from Illumina and Sanger sequencing. Among the 30 isolates, there was 100% concordance for *accA*, *gki*, *hom*, *oppC*, *patB*, *rlmN*, *tsf*, and the corresponding STs (Tables S13 to S19). The Sanger sequencing results therefore demonstrate that the rare alleles defined using the Illumina platform are unlikely sequencing errors (Table S20).

### Epidemiological application of S. mitis typing schemes.

Finally, we investigated the potential epidemiological application of the schemes by analyzing global invasive and carriage S. mitis isolates. The global ML phylogeny showed that the S. mitis isolates that clustered into 259 STs and 258 global sequence clusters were distributed across multiple geographical regions (Fig. S1). Generally, no clear geographical clustering of STs or global sequence clusters was observed, likely due to the extensive S. mitis diversity and the limited data set. Among the invasive isolates, we identified two pairs of infective endocarditis isolates, all from the United Kingdom, belonging to the same sequence type and global sequence cluster, namely, ST30-GSC28 and ST36-GSC27 (highlighted in Fig. S1). However, we did not have patient-level epidemiological data; therefore, it was unknown whether these isolates were from the same individual or are common lineages in the U.K. population.

## DISCUSSION

Here, we present a novel MLST scheme and PopPUNK sequence clusters for S. mitis. Our robust quality control procedure using the bioinformatic taxonomic classification tool (22) ensured that the genomes analyzed belonged to S. mitis. Therefore, the MLST and sequence clustering definitions are based on a well-curated data set. The developed strain typing methods showed high discriminatory power when applied to S. mitis isolates sampled from a cohort of healthy individuals. Crucially, through their integration with the widely used and publicly available MLST and global cluster databases, these genomic schemes pave the way for easily defining new S. mitis strain types (29, 31). Our validated S. mitis MLST PCR primers can also be used together with Sanger sequencing in resource-limited settings to investigate the relatedness of clinical S. mitis strains. The tools developed here will facilitate S. mitis genomic surveillance as done for other species, such as S. pneumoniae (34) and Salmonella enterica (61).

S. mitis is a highly genetically diverse species, sometimes seen as a species complex (13, 62, 63). Therefore, distinguishing different S. mitis strains is critical to understanding its epidemiology, population structure, and evolution within hosts and at the population level. MLST has been able to characterize the considerable population diversity of Helicobacter pylori (64–66), a similarly highly diverse species. Our MLST and PopPUNK global sequence clustering schemes adequately captured the genetic diversity of the species with high resolution. We show that the S. mitis MLST and sequence clustering schemes were highly concordant, and they inferred unique STs and global sequence clusters that highlighted the remarkable genetic diversity of the species. Although our analysis was based on the largest collection available to date, the high genetic diversity revealed in this study of 322 S. mitis genomes represents only the tip of the iceberg.

To assess the utility of the genomic tools in resolving the genetic relatedness of S. mitis isolates, our MLST and global clustering approaches were applied to carriage isolates obtained from 12 healthy individuals. Our results showed a high degree of within- and between-host genetic diversity of S. mitis isolates, with a range of 1 to 5 distinct STs being found within individuals. These results suggest that carriage of multiple S. mitis lineages is common in healthy individuals, and the observed high within-host genetic diversity is similar to that seen in other pathogens, such as H. pylori (67–69). Therefore, our findings emphasize that sampling a single S. mitis isolate from an individual is not sufficient to capture the within-host diversity of the species. The sequence types defined based on the MLST scheme were supported by pairwise SNP distances between strains and phylogeny, and all the isolates that differed by <150 bp clustered together in the phylogeny, shared the same genotypic AMR profiles, and belonged to the same ST. Although the presence of multiple distinct S. mitis lineages among individuals has previously been described using 1 to 4 housekeeping genes (13, 62, 70), the use of a larger number of housekeeping genes in our scheme will provide a higher resolution to detect more cocolonization events. We have also shown that the MLST and PopPUNK schemes can potentially be used together with core genome SNP comparisons to obtain a high discriminatory power that may be sufficient to study the transmission of S. mitis isolates. Three individuals from the prospective pilot carriage study carried ST261 strains belonging to global cluster 4, while ST27 strains belonging to global cluster 11 were identified in 2 individuals. The pairwise SNP distances for the ST261 and ST27 strains ranged between 21 and 23 bp and 11 and 20 bp, respectively, which provided further support for potential transmission, particularly considering the high genetic diversity of the S. mitis species.

Overall, the S. mitis MLST and PopPUNK global sequence clustering schemes developed here offer a robust and standardized approach for molecular typing to identify genetically similar strains at local and global contexts. Both tools are highly portable and publicly available for immediate use and allow for the integration of new genomic data as they become available, making them particularly useful for S. mitis surveillance to better understand disease, carriage, and transmission. Our genomic tools can be integrated into WGS analysis pipelines to investigate population diversity of the S. mitis species.
