# Supplementary material for: Novel Multilocus Sequence Typing and Global Sequence Clustering Schemes for Characterizing the Population Diversity of Streptococcus mitis
Source: J Clin Microbiol. 2022 Dec 14;61(1):e00802-22. doi: 10.1128/jcm.00802-22 (PMC9879099; doi:10.1128/jcm.00802-22)
Supplement: Supplemental file 1 — Fig. S1 to S3. Download jcm.00802-22-s0001.pdf, PDF file, 1.1 MB [file jcm.00802-22-s0001.pdf]

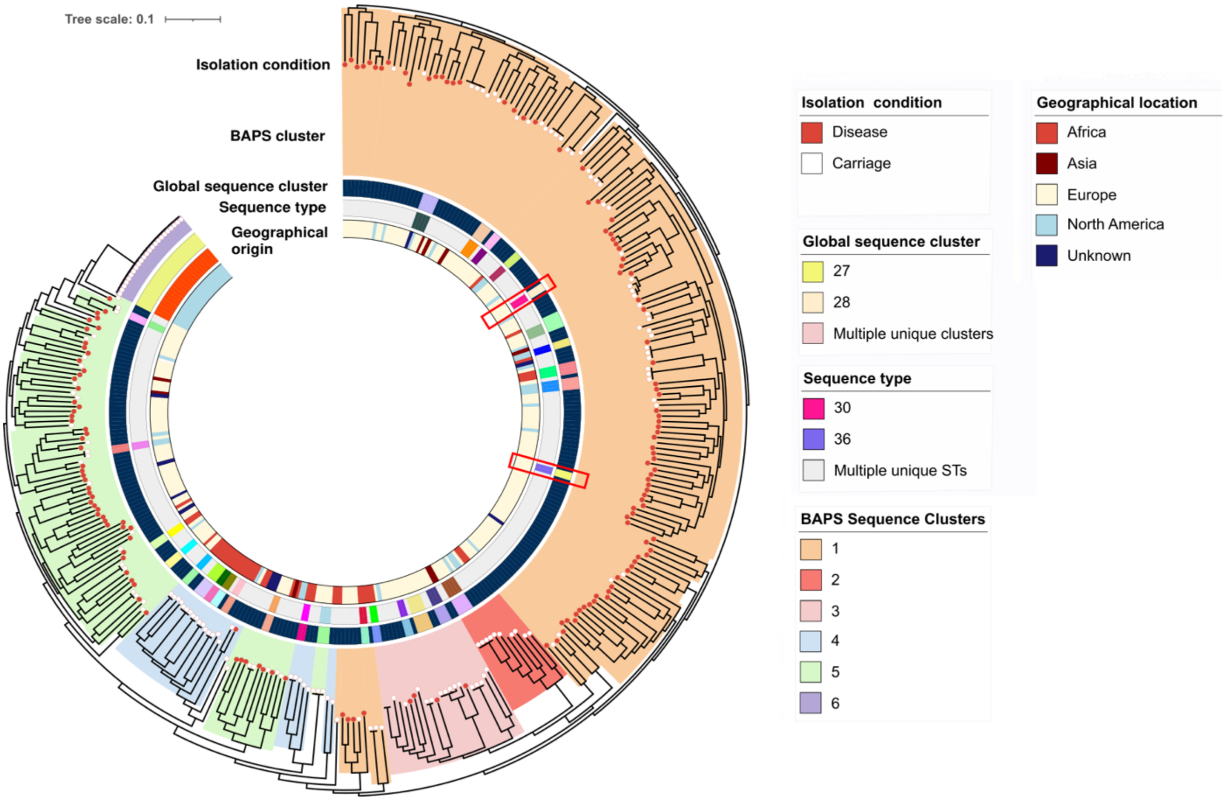

Fig S1: Maximum likelihood core genome phylogeny of global *S. mitis* isolates. The ML phylogeny was constructed using core genome SNPs and the tree was visualised in iTOL. The ML phylogeny demonstrates genetic similarity and diversity among the *S. mitis* isolates. The tree tips show isolates obtained from disease (red), carriage (white), and unknown sources (no symbol). The coloured clades indicate the sequence clusters defined by BAPS. From the inner to outer rings the isolate metadata shows the global sequence cluster defined by PopPUNK, sequence type, and geographical origin.

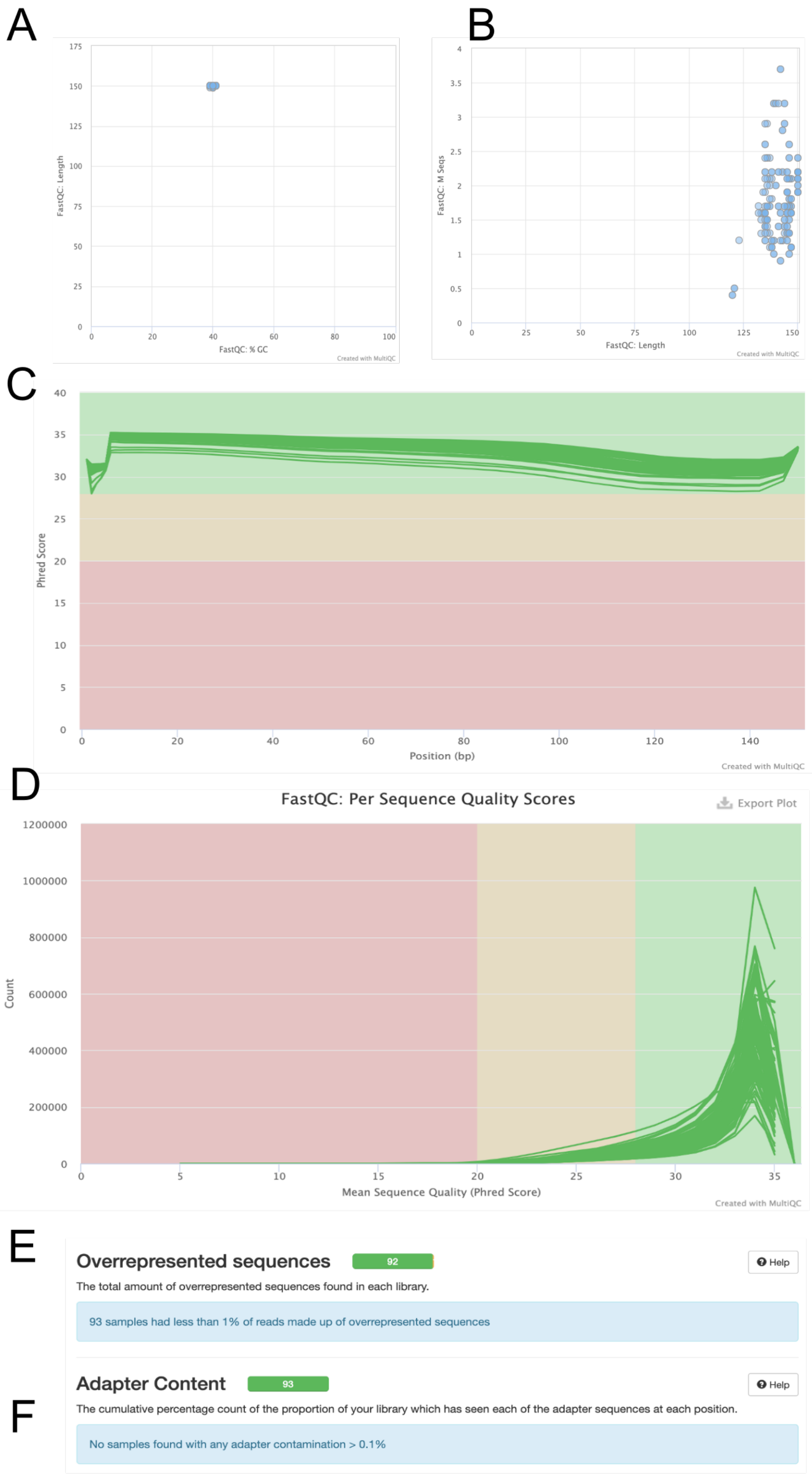

Fig S2: Quality control for 186 sequenced *S. mitis* isolates after read trimming and removal of adapters. The QC data was generated using FastQC for individual files and aggregated into a single file using MultiQC. A) Average sequence read

length (bp) plotted against %GC content B) Number of sequence reads per isolate (Million scale) against average sequence read length (bp) C) Average quality score per base position per sample. Each line represents a sample. Scores greater than 30 (green region) indicate a good quality. D) Average quality score per paired-end reads. Each line represents a sample. Scores greater than 30 (green region) indicate a good quality. E) Total amount of overrepresented sequences per sample. F) Sequencing adapter content per sample.

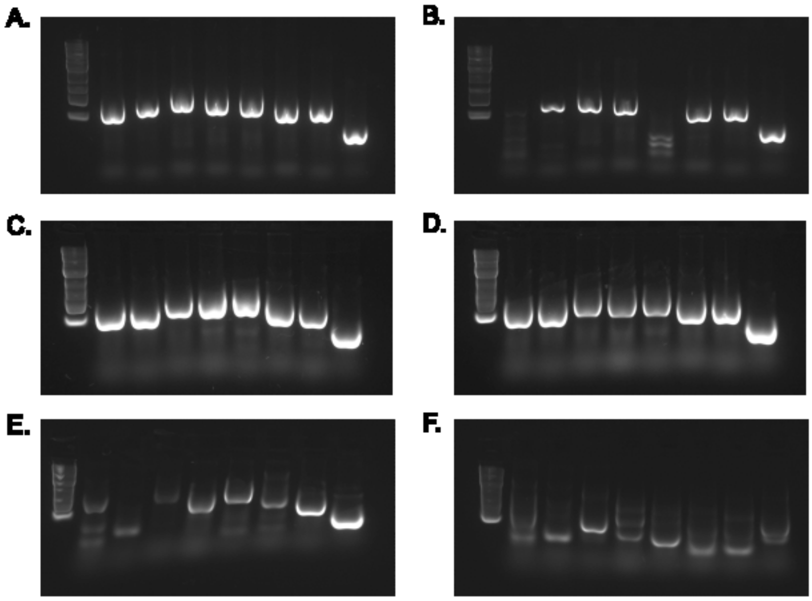

Fig S3. Gel electrophoresis results for MLST primer set testing with (A) *S. mitis* SK142, (B) *S. oralis*, (C) *S. pneumoniae* TIGR4, (D) S*. pneumoniae* BHN418, (E) *S. agalactiae* COH1, and (F) *E. coli* NEB® 5-alpha. The leftmost lane contains NEB 1kb DNA ladder for size comparison. The rightmost lane contains PCR amplification products using universal 16S rRNA primers optimised for *S. pneumoniae* as a control (Bentley *et al.*, 1991)*.* The other 7 lanes contain PCR amplification products using MLST primer sets specific for *S. mitis* (1) *accA,* (2) *gki,* (3) *hom,* (4) *oppC,* (5) *patB,* (6) *rlmN,* and (7) *tsf.* Image brightness/contrast settings were adjusted using FIJI.
